# Hidden fuels: exploring the association between cuticular waxes and leaf flammability in a mediterranean-type forest

**DOI:** 10.3389/fpls.2026.1942980

**Published:** 2026-09-09

**Authors:** Carla Hernández, Melisa Blackhall, Carolina Quintero, Jan Bergmann, Lautaro Taborga, Fabián Guerrero

**Affiliations:** 1 Universidad Técnica Federico Santa María, Department of Mechanical Engineering, Valparaíso, Chile; 2 CONICET-Universidad Nacional del Comahue, INIBIOMA, San Carlos de Bariloche, Argentina; 3 Pontificia Universidad Católica de Valparaíso, Facultad de Ciencias, Instituto de Química, Valparaíso, Chile; 4 Universidad Técnica Federico Santa María, Department of Chemistry, Natural Products Laboratory, Valparaíso, Chile

**Keywords:** cuticular waxes, fire vulnerability, leaf flammability, phytochemistry, plant metabolites, sclerophyllous species

## Abstract

Understanding the chemical drivers of plant flammability is essential for predicting fire behavior in fire-prone ecosystems. While previous research has largely focused on the role of terpenes, the contribution of cuticular waxes to foliar flammability remains poorly understood. In this study, we explored foliar flammability in six native species of the Chilean sclerophyllous forest, five of which were successfully characterized for cuticular wax chemistry. Foliar flammability was characterized by measuring ignition probability, ignition time, flame duration, and combustion time of foliar samples, and it was related to the chemical profile of cuticular waxes, including compound composition and concentrations. Results showed that higher total wax loads were associated with increased flammability, particularly through shorter ignition times and prolonged combustion. Non-alkane compounds, especially ketones and terpenoids, emerged as the main components associated with flammability. In particular, 16-hentriacontanone was consistently associated with highly flammable species, suggesting a potential association with ignition-related flammability. Among alkanes, nonacosane was correlated with flame duration. Our results highlight the importance of exploring the relationships among chemical compound classes potentially associated with flammability. This study provides the first evidence that non-alkane constituents of cuticular waxes appear to be more strongly associated with flammability than alkanes. By expanding the focus beyond terpenes, our findings offer a novel perspective on the chemical basis of plant foliar flammability and contribute to improving fire-risk assessment in Mediterranean-type ecosystems under increasing drought and fire activity.

## 1 Introduction

Wildfires represent an escalating threat worldwide. The convergence of global climate change with multiple anthropogenic drivers has led to marked increases in wildfire frequency and severity, generating profound ecological, social, and economic impacts (Ellis et al., 2022; Clarke et al., 2025). In Chile, this trend has been tragically illustrated by the 2017 wildfire season, during which more than 500,000 ha of forested land burned, and more recently by the February 2024 megafires in the Valparaíso region (Pliscoff et al., 2020; Martínez et al., 2024). These events rank among the deadliest wildfires globally over the last century, starkly exposing the vulnerability of wildland–urban interface areas. Mediterranean-type ecosystems are inherently prone to wildfires, particularly during the summer, when low precipitation and high temperatures promote vegetation desiccation and increase fuel flammability (Keeley et al., 2012). Central and south-central Chile has a Mediterranean climate that supports the Chilean Mediterranean Sclerophyll Forest, a global biodiversity hotspot distinguished by high levels of endemism (Cruz et al., 2013). In this region, as in the other four well-known fire-prone Mediterranean-type ecosystems (i.e. the Mediterranean Basin, California, southwestern South Africa, and southwestern Australia), high population density, land-use change, and ongoing climatic shifts pose severe threats, with habitat loss and wildfire risk emerging as the dominant pressures (Keeley et al., 2012; Cruz et al., 2013). Climate change projections for Chile indicate that these conditions are likely to intensify in the coming decades, further elevating wildfire risk (Urmeneta et al., 2021). In this context, understanding the drivers that modulate wildfire risk and severity becomes increasingly critical.

Within the fire behavior framework, vegetation constitutes one of the three components of the fire triangle, along with weather and topography, but it is the only component that can be directly managed or modified to reduce wildfire risk (McLauchlan et al., 2020). Consequently, the contribution of vegetation to fire behavior warrants careful examination, as fuel traits offer a tangible leverage point for mitigation strategies, in contrast to largely uncontrollable climatic drivers. Flammability, defined as the susceptibility of vegetation to ignite and sustain combustion, is a fundamental property governed by the physicochemical characteristics of plant fuels (Pausas et al., 2017). Among physical fuel properties, traits such as fuel moisture content, surface-area-to-volume ratio, leaf thickness, bulk density, or fuel packing are strongly associated with ignition probability and fire behavior, by modulating heat transfer, drying rates, or oxygen availability during combustion (Archibald et al., 2018). Chemical properties of fuels have gained recent attention, with phytochemicals such as terpenes and other essential oils being the most frequently cited compounds linked to increased foliar flammability due to their high volatility and energetic content (e.g., Ganteaume et al., 2021; Guerrero et al., 2022). However, recent research in the Chilean Mediterranean ecosystem highlights that foliar flammability cannot be fully explained by terpene content alone (Guerrero et al., 2024). In this recent study, the flammability of several native sclerophyll species was strongly influenced by other classes of secondary metabolites, including ketones and aldehydes, whose concentrations highly correlated with foliar flammability.

To withstand sustained abiotic stress, such as drought and heat waves, sclerophyllous plants have evolved complex physiological and biochemical adaptations that enhance survival and functional efficiency (Peguero-Pina et al., 2020). Among these strategies, the synthesis and accumulation of secondary metabolites, including essential oils (terpenes), play critical roles in plant defense and environmental adaptation (Lambers et al., 2008). Various studies have proposed that these compounds can substantially increase foliar flammability and wildfire risk due to their high energy content and low ignition temperatures (Ormeño et al., 2009; Pausas et al., 2016; Della Rocca et al., 2017). Under wildfire conditions, terpenes volatilize rapidly, generating an atmosphere enriched in combustible gases that promotes foliage ignition and facilitates rapid flame propagation. Another strategy is the synthesis and accumulation of cuticular waxes, but this aspect of plant chemistry has remained largely unexplored in relation to flammability.

Cuticular waxes form a protective coating over the leaf epidermis and have evolved as a key adaptive feature enabling plants to tolerate a wide range of biotic and abiotic stresses (Lambers et al., 2008). Cuticular waxes constitute an important reservoir of long-chain aliphatic compounds (typically C21–C35), including mainly fatty acids and their derivatives, such as alkanes, primary and secondary alcohols, aldehydes, ketones, and esters, which accumulate on the outermost surface of leaves (Samuels et al., 2008). Among wax constituents, long-chain n-alkanes (linear alkanes) are especially relevant in the context of flammability because they are ubiquitous in vascular plants (Samuels et al., 2008) and are relatively stable, combustible compounds with physicochemical properties that can facilitate ignition, including relatively low flash points (Rumble, 2025). In contrast, non-alkanes encompass chemically heterogeneous compounds that differ substantially in volatility, flash point, thermal stability, and heat of combustion, and therefore their contribution to flammability is not expected to be equivalent. Among these, terpenoids and some oxygenated compounds, such as aldehydes and ketones, may contribute to flammability through their volatility and/or the generation of volatile products during thermal degradation, potentially facilitating ignition under intense heating (Pausas et al., 2016; Greenberg et al., 2006). Moreover, the presence of these chemical groups in leaf tissues has been associated with leaf flammability in Chilean Mediterranean vegetation (Guerrero et al., 2024).

Beyond their potential role in combustion processes, cuticular waxes are multifunctional, contributing to protection against UV radiation, pathogens, herbivores, extreme temperatures, high salinity, and regulation of permeability barriers. However, their most critical ecophysiological function is to limit cuticular transpiration and reduce non-stomatal water loss (Lambers et al., 2008); making them very relevant in Mediterranean-type vegetation.

In addition to their constitutive synthesis, evidence indicates that the chemical composition of leaf cuticular waxes is closely linked to environmental variability, reflecting plant strategies to cope with changing conditions. For instance, drought stress has been shown to induce marked changes in both the quantity and composition of cuticular waxes across diverse plant species (Shepherd and Wynne Griffiths, 2006). These responses can involve increases in total wax content and shifts in metabolite profiles that reduce water loss and enhance drought tolerance (Ormeño et al., 2020). To date, the study by Ormeño et al. (2020) remains the only investigation explicitly addressing the relationship between plant flammability and physicochemical traits associated with cuticular waxes. Focusing on leaf litter of *Quercus coccifera*, a dominant shrub in Mediterranean sclerophyll forests, the authors examined alkane concentrations in cuticular waxes under conditions of prolonged experimental drought (5.5 years of reduced precipitation). As expected, higher concentrations of cuticular alkanes, specifically pentacosane, hexacosane, heptacosane, octacosane, nonacosane, triacontane, and untriacontane, achieved under intensified drought conditions, were associated with increased litter-bed flammability. Based on these findings, the authors suggested that the flammability of *Q. coccifera* is likely to increase under drier future Mediterranean climates (Ormeño et al., 2020).

In this context, cuticular waxes may reach particularly high concentrations under arid conditions, such as those characterizing Mediterranean summers, especially during prolonged droughts, potentially enhancing leaf flammability. However, despite their well-established role in plant water balance and stress tolerance, the contribution of cuticular waxes to the flammability of live fuels remains largely unexplored, particularly in native Mediterranean-type ecosystems. Here we explored the relationship between leaf flammability and the chemical traits of cuticular waxes in six native woody species of the Chilean sclerophyll forest. Specifically, we quantified flammability parameters under controlled laboratory conditions and characterized the composition and concentration of cuticular wax compounds using gas chromatography–mass spectrometry, and then integrated these datasets to identify the chemical traits most strongly associated with fire susceptibility. We hypothesized that leaf flammability would be associated with both total cuticular wax concentration and its chemical composition, with specific chemical classes and individual compounds potentially contributing disproportionately to flammability. In particular, we expected higher total wax content, as well as higher content of alkanes and chemically reactive and/or volatile non-alkane compounds, such as aldehydes, ketones, and terpenoids, to be associated with increased leaf flammability. By exploring associations between cuticular wax chemistry and flammability traits, this study provides a first step toward understanding the potential role of leaf surface chemistry in shaping flammability patterns in Mediterranean-type ecosystems.

## 2 Methods

### 2.1 Study area

The study area is located in central Chile, one of the five Mediterranean-climate regions worldwide and a global conservation priority due to its high levels of plant endemism (Niemeyer et al., 2002; Keeley et al., 2012). The regional climate is Mediterranean, characterized by marked seasonality with cool, wet winters and hot, dry summers. Mean annual precipitation is approximately 700 mm, and mean annual temperature is around 11 °C (Sarricolea et al., 2017). The study was conducted in Río Clarillo National Park, located in the municipality of Pirque, Metropolitan Region of Chile (approximately between 33°41′ and 33°52′ S and 70°28′ and 70°37′ W). The park spans a wide altitudinal range, from roughly 850 to 3,000 m.a.s.l., encompassing strong environmental gradients that influence vegetation composition. Río Clarillo National Park represents one of the most important protected areas of the central Chilean Andes ecosystem (Teillier Arredondo et al., 2005). The reserve covers 9,260 ha and is dominated by a heterogeneous vegetation mosaic that includes sclerophyllous and thorny shrublands, as well as Andean steppe at higher elevations (CONAF, 1996). Although forest plantations of both native and exotic species are also present within the park (Teillier Arredondo et al., 2005), the most representative woody species of the native sclerophyll forest include *Lithraea caustica, Cryptocarya alba, Quillaja saponaria*, and *Kageneckia angustifolia.*

### 2.2 Sample collection

Leaves were collected between 8 and 12 January 2024 from individuals of each of the following native woody species: *Colliguaja odorifera* (Euphorbiaceae), *Cryptocarya alba* (Lauraceae), *Escallonia pulverulenta* (Escalloniaceae), *Lithraea caustica* (Anacardiaceae), *Persea lingue* (Lauraceae), and *Quillaja saponaria* (Quillajaceae). These species were selected because they are among the most abundant and representative woody species of the Mediterranean sclerophyll forest in Río Clarillo National Park (Teillier Arredondo et al., 2005). Within the study area, ten adult individuals per species were randomly sampled, maintaining a minimum separation of 5–20 m among individuals, and up to 150 m among sampled plant groups. Sampling was conducted between 893 and 973 m a.s.l, on a southwest-facing slope. In total, approximately 80 g of fresh leaf material was collected per individual, following the protocol described by Guerrero et al. (2021, 2024). Briefly, only fresh and healthy leaves were selected, avoiding any material showing signs of physical damage, disease, or herbivory. Leaves were collected and pooled from three canopy positions differing in sun exposure (upper, middle, and lower crown) to ensure representative and homogeneous samples. Each sample was placed in a sealed plastic bag and transported to the laboratory in a portable cooler maintained at −4 °C until further processing. Once in the laboratory, the collected plant material was divided into three subsamples. One subsample was used for the determination of leaf moisture content, which was determined by oven-drying 10 g of fresh leaves at 60 °C for 48 h and calculated as [(wet mass − dry mass)/dry mass] × 100 (Guerrero et al., 2024). A second subsample was used for determining flammability parameters, while the third was reserved for chemical characterization and quantification of cuticular waxes. Thus, fuel moisture content was determined from a separate subsample and was not measured on the same leaf material used in the flammability assays. Accordingly, FMC values were used to characterize the general hydration status of the sampled foliage rather than as individual-level covariates of flammability.

### 2.3 Flammability measurements

Flammability of the plant samples was assessed using a 500 W epiradiator (model 534 RC2, Quartz Alliance, France), a standard device in flammability studies that provides controlled and reproducible thermal conditions for combustion experiments (Ocampo-Zuleta et al., 2024). For each individual, 20 successive combustion tests were conducted using 20 samples, weighing 1.0 ± 0.1 g each. Following the protocol described by Guerrero et al. (2024), each sample was placed on the epiradiator surface, which was maintained at approximately 440 °C. A pilot flame positioned 4 cm above the epiradiator surface facilitated the ignition of volatile compounds released during the thermal degradation of the plant material. During each trial, four flammability parameters were recorded. First, the probability of ignition (%) was calculated as the proportion of trials in which a flame was observed. Samples were classified as non-ignited when no visible flame developed after placement on the epiradiator surface. Nevertheless, these samples were allowed to continue smoldering until the complete disappearance of glowing embers, ensuring that the entire combustion process was completed and combustion time could be recorded. Second, the ignition time (s) was defined as the time elapsed between placement of the sample on the epiradiator surface and the appearance of a flame. Third, flame duration (s) was recorded as the time that the flame was sustained; and finally, combustion time (s) was defined as the time from initial exposure of the leaf to heat until the complete disappearance of glowing embers, capturing the full thermal degradation of the sample, including the smoldering phase (Guerrero et al., 2024).

### 2.4 Chemical extraction, identification, and quantification of cuticular waxes

A subsample of 30 g of fresh leaves was used for cuticular wax analysis, following a modified methodology based on previous leaf wax extraction protocols (Cerda-Peña and Contreras, 2022). The plant material was air-dried at room temperature, avoiding direct solar radiation. Cuticular waxes were extracted using a microwave digestion system (MILESTONE, ETHOS X; serial number: 23047263). Leaves were kept intact (i.e., not cut) to preserve their natural morphology and were placed individually into 100 mL glass tubes with 30 mL of hexane. A composite sample of multiple intact leaves per individual was prepared, using the number of leaves necessary to obtain 1 g of dry mass. The microwave extraction was performed under the following conditions: a heating phase of 5 min from 30 °C to 50 °C at a microwave power of 950 W, followed by a holding phase at 50 °C for 20 min, and a final cooling phase of 15 min until room temperature was reached. Thus, the total extraction time was 40 min. After extraction, the solvent extracts were concentrated using a rotary evaporator and stored at 3 °C until subsequent analysis. During storage, precipitate formation was observed in some extracts; therefore, all samples were filtered using a Büchner funnel fitted with qualitative filter paper (150 mm diameter; Cat. No. 1001-150) before analysis. Filtered extracts were analyzed using a gas chromatograph coupled to a mass spectrometer (GC–MS; GCMS-QP2010 Ultra, Shimadzu, Kyoto, Japan).

Individuals were randomly assigned to composites, meaning that the ten individual extracts per species were combined into three composite samples per species (mixing 500 µL per individual): two consisting of three individuals each and a third comprising the remaining four individuals. Hence, each individual contributed to only one composite sample. Thus, the three composites were derived from non-overlapping sets of individuals and represented biological composite samples rather than technical replicates. This approach allowed us to retain some biological replication for chemical characterization while keeping the analytical costs and processing time required for GC-MS analysis feasible.

From each mix, 1 µL was then taken for GC-MS injection. Separations were performed on a non-polar fused silica capillary column (Rxi-5Sil MS; 15 m × 0.25 mm i.d., 0.25 µm film thickness with a 10 m guard column; Restek, Bellefonte, PA, USA). Helium was used as the carrier gas at a constant linear velocity of 40 cm s-1. The oven temperature program was as follows: initial temperature of 100 °C held for 1 min, increased to 200 °C at a rate of 20 °C min^−1^, and then raised to 330 °C at 10 °C min^−1^, where it was held for 30 min. The total run time was 49 min. Samples were injected in split mode (1:10), and the injector temperature was set at 220 °C. The mass spectrometer operated in full scan mode with an m/z range of 35–500, a scan rate of 0.3 s per scan, and an electron impact ionization energy of 70 eV. Chemical compounds were tentatively identified using GCMS Postrun Analysis software by comparing mass spectra and retention indices with those reported in the NIST11 mass spectral library and in the NIST Chemistry Webbook (NIST Mass Spectrometry Data Center, 2026). For compound quantification, 1-octadecanol (≥ 99.9%; Sigma-Aldrich) was used as an external standard. Initially, 1.1 mg of 1-octadecanol was dissolved in 1.1 mL of hexane to prepare a set of standard solutions (0.3, 1, 3, 10, 30, and 50 μg mL-1) and generate a calibration curve by plotting peak area vs. concentration. The linear regression was used to calculate the concentrations by using the respective peak areas of the compounds in the samples. It is important to note that *E. pulverulenta* was excluded from chemical analyses because its extracts showed extensive co-elution and an unresolved complex mixture (UCM), preventing reliable identification and quantification of individual compounds. Attempts to improve chromatographic resolution by modifying the oven temperature program were unsuccessful, and we therefore excluded this species rather than applying a species-specific derivatization or analytical protocol that would compromise comparability among species.

### 2.5 Data analysis

Differences among species in flammability parameters (probability of ignition, ignition time, flame duration, and combustion time), and in major classes of cuticular wax compounds (total wax content, alkanes, non-alkane compounds), were assessed using linear (LMs) and generalized linear models (GLMs), with species as a fixed factor. Analyses were conducted in R (v. 4.4.0). Pairwise comparisons were conducted using Tukey-adjusted contrasts (packages emmeans and multcomp). Model assumptions were evaluated using Shapiro–Wilk tests and visual inspection of residuals. Most variables met normality assumptions; alkanes and non-alkanes concentration were modeled using a Gaussian distribution with a log link function (glmmTMB); and probability of ignition was analyzed using a binomial distribution. A continuity correction (± 0.5) was applied to proportional data before *post hoc* tests when 0% or 100% values were present. Possible associations between species and chemical compounds were evaluated using the Indicator Value Index (IndVal), which identifies compound groups characteristic of each species based on exclusivity and fidelity (abundance matrix), with significance assessed via permutation tests (p < 0.05).

Two Principal Component Analyses (PCA) were used to summarize and explore variation in flammability variables and chemical composition, using the correlation matrix based on Pearson correlation coefficients. This allowed the assessment of interspecific variation in all traits simultaneously in order to detect similarities or differences across species. PCAs were conducted using PAST software (v. 5.2, 2025). For all analyses, components with eigenvalues > 1 were retained, following the Kaiser–Guttman criterion, and PCs interpreted according to the contribution of individual variables. Bartlett’s test of sphericity was used to confirm the suitability of the data for dimensionality reduction (p < 0.05). The first PCA included the four flammability variables across 60 individuals, corresponding to the six species. The first principal component (PC1) was interpreted as a flammability gradient based on variable loadings, and species mean PC1 scores were used to corroborate species clustering. The second PCA was performed on mean chemical compound concentrations (alcohols, aldehydes, alkanes, alkenes, ketones, esters, terpenoids, and unidentified compounds), excluding *E. pulverulenta* due to chromatographic limitations. Following this chemical PCA, a third additional flammability PCA was conducted on the same reduced chemical dataset (i.e., excluding *E. pulverulenta* and averaged composite samples) to ensure direct and balanced comparability.

Relationships between flammability and chemical composition were explored using Pearson correlation analyses, with each composite sample representing one observation and paired with the mean flammability values of the same individuals contributing to that composite. Correlations were calculated between PC1 of flammability and PC1 of chemical composition, as well as between individual flammability variables and the concentrations of chemical groups and selected compounds. Because three composite samples were obtained from each of the five species, these analyses were considered exploratory and were used to identify broad associations between chemical composition and flammability rather than to infer causal or general species-level relationships.

## 3 Results

### 3.1 Flammability differences across species

**Figure 1:**
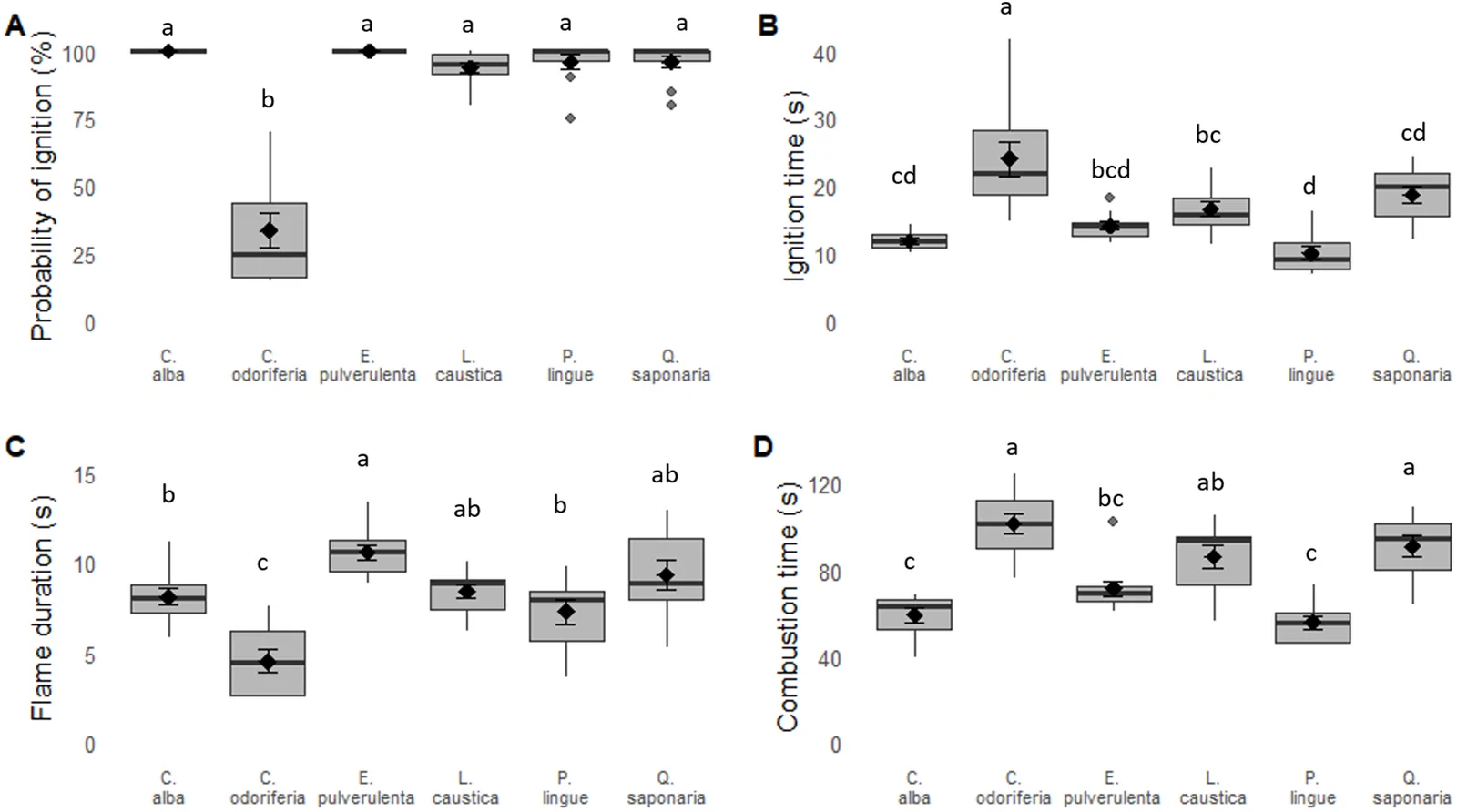
Mean probability of ignition **(A)**, ignition time **(B)**, flame duration **(C)**, and combustion time **(D)** for six native species from the Chilean sclerophyll forest. Boxes represent the interquartile range (IQR), horizontal lines indicate medians, whiskers extend to 1.5 × IQR, diamonds indicate means, and error bars represent ±1 SE. Different letters denote significant differences among species (p < 0.05).

The probability of ignition varied markedly among species. *C. odorifera* showed a substantially lower probability of ignition (33%), whereas the remaining species exhibited consistently high probabilities (98–100%; χ² = 389.3, df = 5, 54, p < 0.0001; **Figure 1a**). Considering only samples that ignited, time to ignition also differed significantly among species (F = 13.92, df = 5, 54, p < 0.0001; **Figure 1b**), with *P. lingue* showing the shortest time to ignition and *C. odorifera* the longest. Flame duration varied significantly across species (F = 12.23, df = 5, 54, p < 0.0001; **Figure 1c**), with mean values ranging from 4.6 to 10.6 s. *C. odorifera* exhibited a significantly shorter flame duration than all other species. Total combustion time also differed among species (55.8 s; F = 19.1, df = 5, 54, p < 0.0001; **Figure 1d**), with *C. odorifera* showing the longest mean combustion time (101.7 s), whereas *P. lingue* exhibited the shortest. Fuel moisture content did not vary across species (mean all species 133 ± 1.78%; F = 0.86, df = 5, 49, p = 0.51).

Pearson correlation analysis considering the six species revealed several significant relationships among flammability parameters (**Figure A1**). Ignition time was positively correlated with combustion time (0.86), but negatively correlated with both probability of ignition (-0.64) and flame duration (-0.45; all p < 0.001). Probability of ignition was positively correlated with flame duration (0.56) and negatively correlated with combustion time (-0.54; all p < 0.001). Fuel moisture content was not significantly correlated with any flammability parameter.

**Figure 2:**
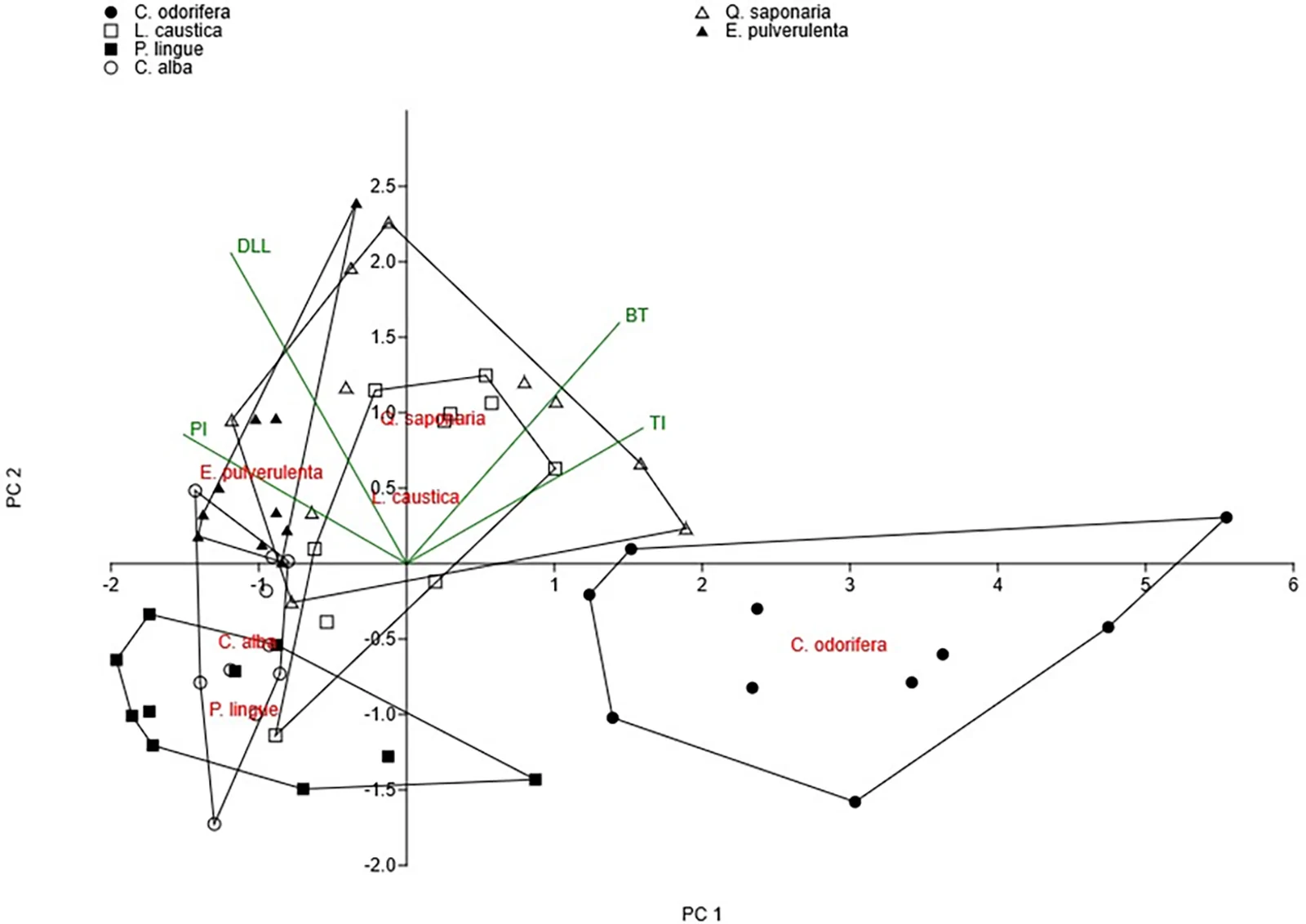
Principal components analysis (PCA) of four measured variables, including: probability of ignition (PI), ignition time (IT), flame duration (FD), combustion time (CT), considering the individuals of the six species analyzed from the Chilean sclerophyll forest. Symbols in the graph indicate each individual, while species labels indicate the mean species groups’ position in the spatial ordination, including convex hulls for each species.

Principal component analysis (PCA) of flammability traits showed that the first two axes explained 90.3% of the total variance (Bartlett’s test of sphericity, p < 0.001; **Figure 2**). PC1 accounted for 67.9% of the variance and was positively associated with ignition time (loading = 0.55) and combustion time (0.50), and negatively associated with probability of ignition (−0.52) and flame duration (−0.41). Thus, higher PC1 values indicated lower flammability (lower ignition probability, longer ignition times, and shorter flame duration), whereas lower values indicated higher flammability. PC2 explained 22.4% of the variance and was positively related to all flammability traits, with the strongest contributions from flame duration (0.71) and combustion time (0.55), reflecting longer flaming and combustion phases. The PCA ordination separated species according to their flammability. *P. lingue* and *C. alba* were the most flammable species, characterized by high ignition probability, short ignition times, long flame duration, and relatively short combustion time. *E. pulverulenta* also exhibited high flammability, although slightly lower than that of *P. lingue* and *C. alba*. *L. caustica* and *Q. saponaria* showed intermediate flammability; although *Q. saponaria* had intermediate PC1 values, it consistently exhibited positive PC2 scores driven by longer flame durations. In contrast, *C. odorifera* formed a distinct group as the least flammable species, with low ignition probability, long ignition times, and short flame duration. This pattern was supported by species-level mean PC1 scores.

To enable direct comparison between flammability traits and chemical composition (see below), a second PCA was performed, excluding *E. pulverulenta*. The results were consistent with the full analysis. PC1 and PC2 explained 93.6% of the total variance (Bartlett’s test of sphericity, p < 0.001), accounting each PC for 75.4% (PC1) and 18.2% (PC2) (**Supplementary Figure A2**). PC1 was positively associated with ignition (loading = 0.54) and combustion (0.49) times and negatively associated with probability of ignition (−0.52) and flame duration (−0.44), representing a gradient from rapidly igniting species to those with delayed ignition and longer combustion. PC2 was mainly driven by flame duration (0.68) and combustion time (0.57). Species distribution along the axes mirrored the full PCA: *P. lingue* and *C. alba* were the most flammable species, *Q. saponaria* and *L. caustica* showed intermediate flammability, and *C. odorifera* remained clearly separated as the least flammable species.

### 3.2 Cuticular wax composition and chemical profiles across species

**Figure 3:**
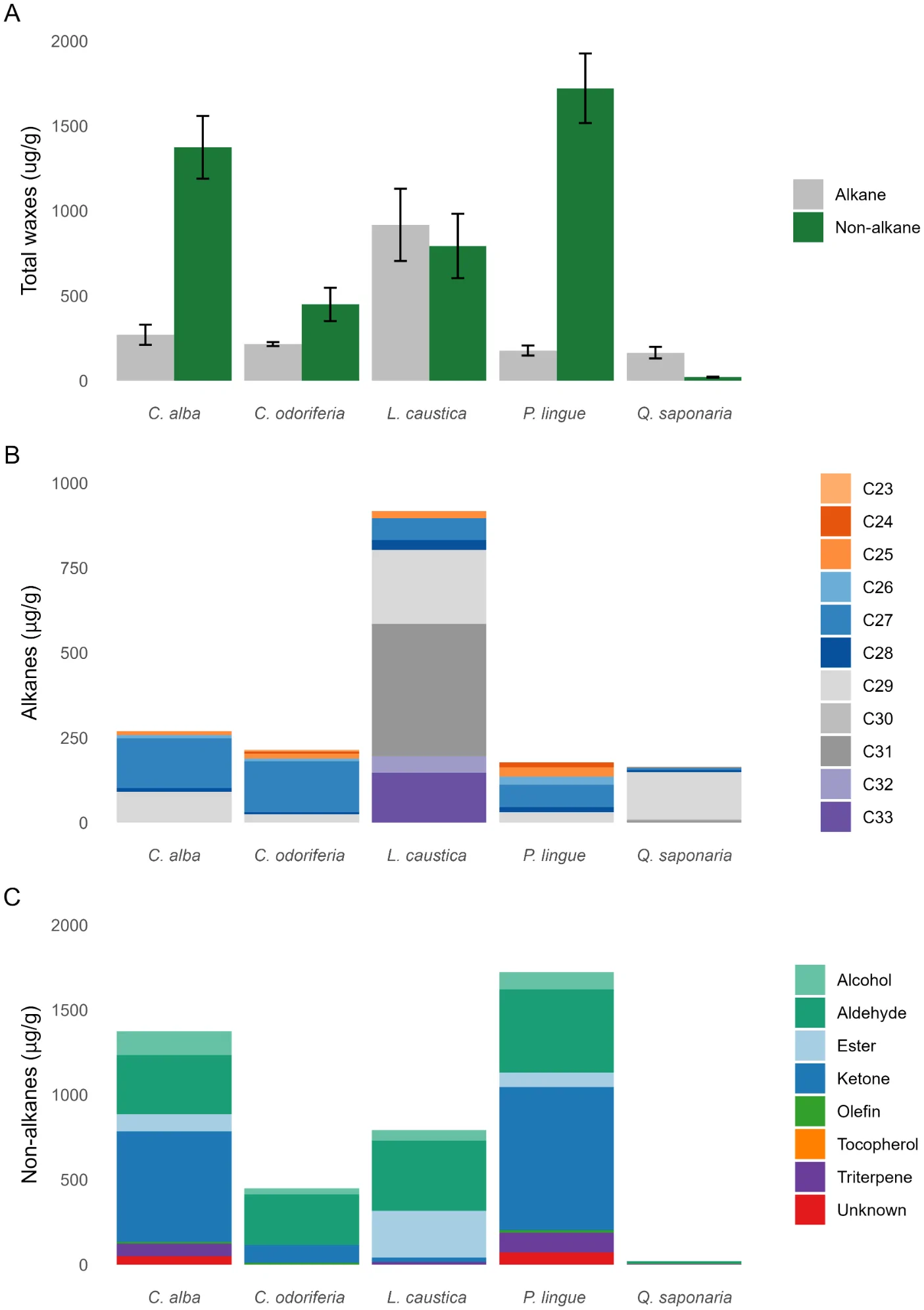
Phytochemical characterization of cuticular waxes in five native species from the Chilean sclerophyll forest, showing **(a)** mean total wax content (μg g^−1^) ± 1 SE, separated into total alkanes and non-alkane total content; **(b)** mean concentrations of individual alkane compounds (μg g^−1^); and **(c)** mean concentrations of non-alkane compounds (μg g^−1^).

Chemical analyses of cuticular waxes revealed several major families of organic compounds. Species differed significantly in total wax content (F = 10.61, df = 4, 10, p = 0.0013; **Figure 3a**), as well as in alkane (F = 45.23, df = 4, 9, p = 0.00001; **Figure 3b**) and non-alkane content (F = 36.89, df = 4, 9, p = 0.00001; **Figure 3c**). *Q. saponaria* and *C. odorifera* showed the lowest total wax contents. In *Q. saponaria*, waxes were strongly dominated by alkanes (89%), whereas *P. lingue* and *C. alba* exhibited the highest proportions of non-alkane compounds (91% and 84%, respectively).

Across species, alkanes, ketones, and aldehydes showed the highest average concentrations. Alkanes, aldehydes, and alcohols were present in all species, whereas ketones were absent only in *Q. saponaria*. Considering alkanes, between five and seven alkane compounds were identified per species, and *L. caustica* was the species with the highest total alkane content. *C. odorifera* was the only species in which tricosane was detected, although at low concentrations. Notably, within ketones, 16-hentriacontanone was abundant in *P. lingue* and *C. alba*, present at lower concentrations in *L. caustica*, and absent in *Q. saponaria* and *C. odorifera*. Aldehydes represented an important fraction of waxes in *C. alba* (21%), *C. odorifera* (45%, mainly octacosanal), *L. caustica* (24%, mainly octacosanal), and *P. lingue* (26%). Alkanes were also abundant, particularly in *Q. saponaria* (89%, with nonacosane alone representing 75%), *C. odorifera* (32%, mainly heptacosane), and *L. caustica* (54%, mainly hentriacontanone and nonacosane). In *P. lingue* and *C. alba*, ketones accounted for nearly half of the wax composition (44% and 40%, respectively), largely driven by 16-hentriacontanone. *L. caustica* showed the most chemically diverse profile, with 26 compounds identified, whereas *P. lingue* and *C. alba* exhibited highly similar chemical profiles (**Figure 3**).

**Figure 4:**
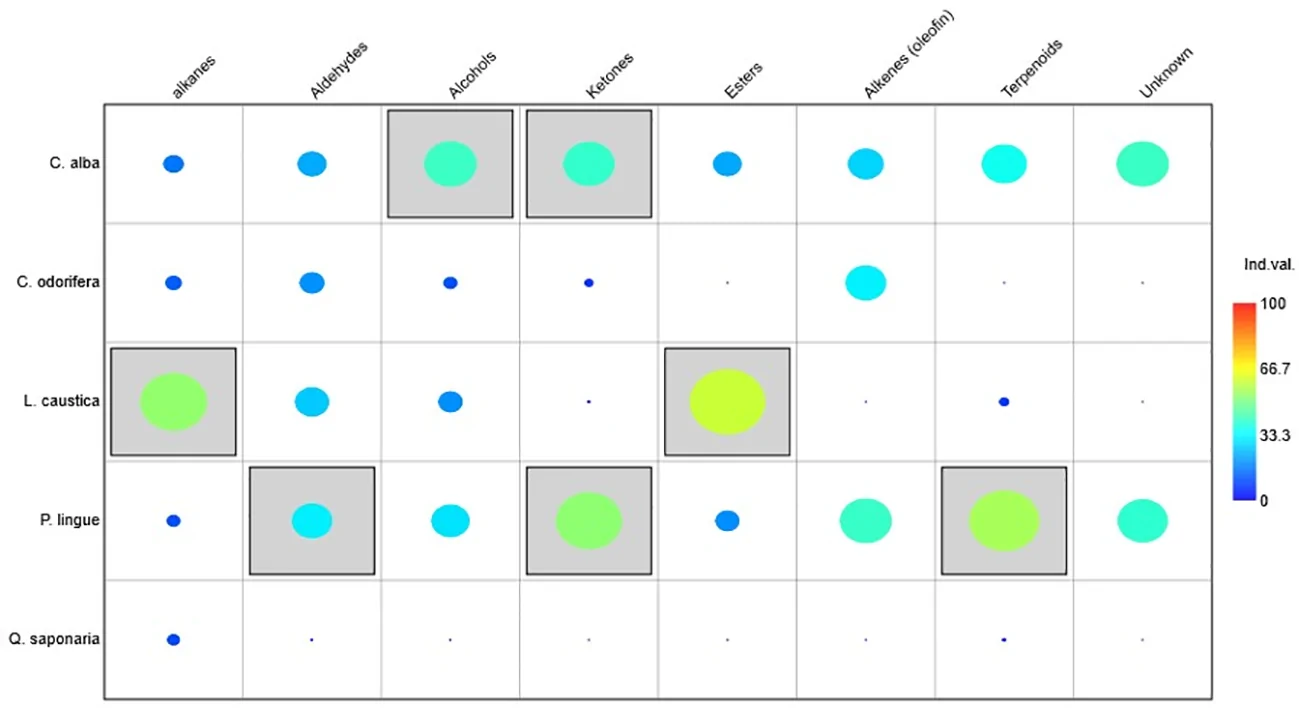
Indicator compound analysis (IndVal) identifying chemical compound groups characteristic of five native species from the Chilean sclerophyll forest based on exclusivity and fidelity. Boxed cells denote significant associations (p < 0.05).

Indicator compound analysis (IndVal) showed that *P. lingue* and *C. alba* —the species with the highest wax content— were mainly characterized by non-alkane compounds: in both species, ketones were the main indicator compounds, and also with aldehydes and terpenoids associated with *P. lingue* and alcohols with *C. alba* (**Figure 4**). *L. caustica* was characterized by alkanes and esters, whereas *C. odorifera* and *Q. saponaria* showed no indicator compounds among the wax groups.

**Figure 5:**
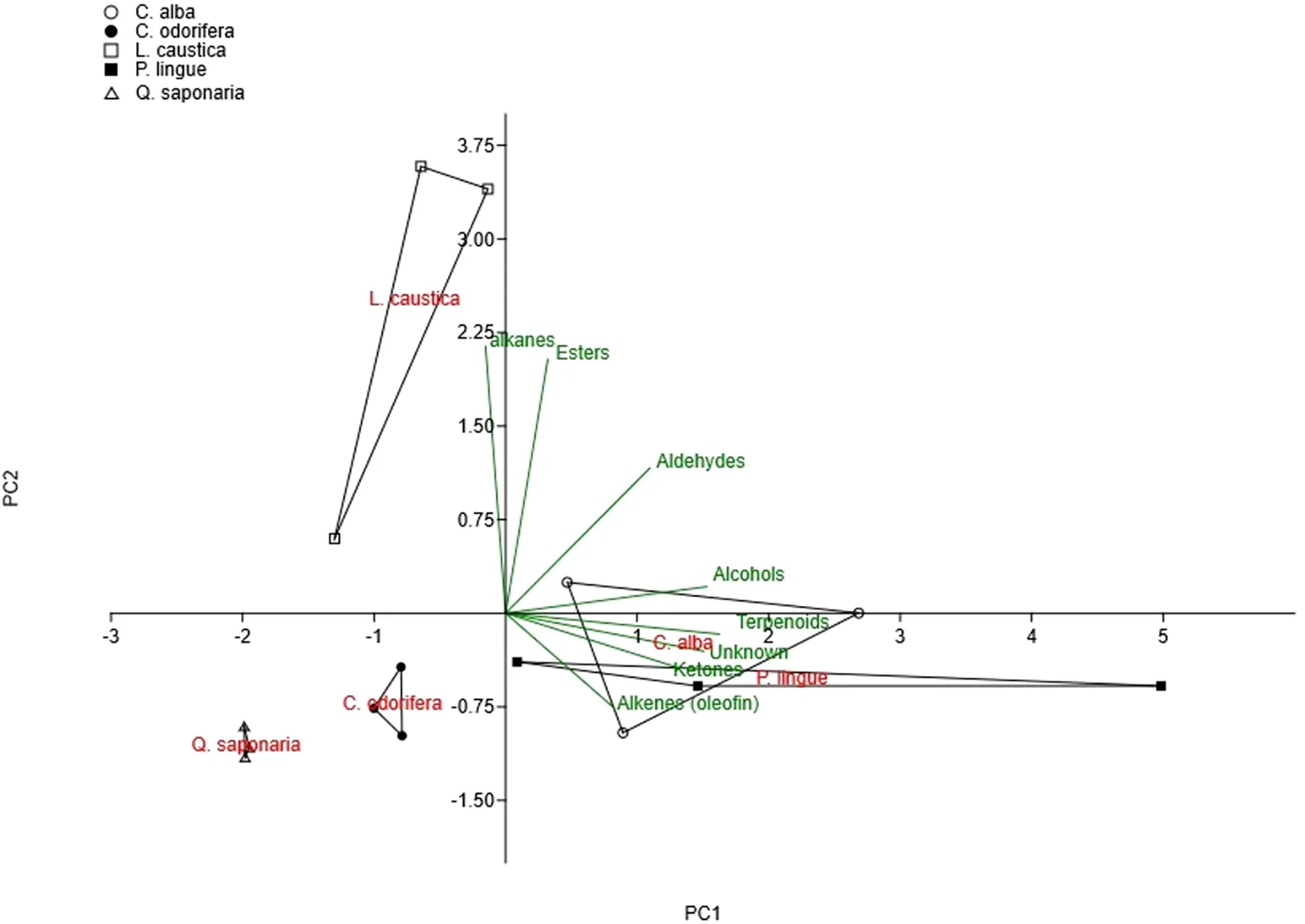
Principal component analysis (PCA) of eight chemical compound groups (alkanes, alkenes, alcohols, aldehydes, esters, ketones -including 16-hentriacontanone- terpenoids including tocopherols and triterpenes, and unknown compounds) measured in individuals of five native species from the Chilean sclerophyll forest. Symbols represent individual samples, whereas species labels indicate mean species positions in the ordination space. Convex hulls encompass individuals of each species.

Principal component analysis of wax chemical composition showed that the first two components explained 73.98% of the total variance (Bartlett’s test of sphericity, p < 0.001; **Figure 5**). PC1 accounted for 45.77% of the variance and was positively associated with terpenoids (loading = 0.49), unknown compounds (0.45), alcohols (0.46), ketones (0.40), and aldehydes (0.33). PC2 explained 28.21% of the variance and was mainly related to alkanes (0.64) and esters (0.61). The ordination separated species: first, *P. lingue* and *C. alba* clustered together, reflecting similar wax compositions dominated by terpenoids (particularly triterpenes, which were the most abundant among this group), ketones, aldehydes, and alcohols; second, *Q. saponaria* and *C. odorifera* were characterized by low concentrations of these compounds; and third, *L. caustica* occupied a distinct position associated with higher abundances of alkanes and esters.

### 3.3 Relationships between flammability and cuticular wax traits

Correlation analyses between flammability and chemical variables revealed several notable patterns (**Supplementary Figures A3**, **A4**). PC1 flammability and PC1 chemistry showed a potential trend (r = −0.47; p = 0.078), where higher concentrations of compounds such as terpenoids, alcohols, aldehydes, and ketones could be associated with increased flammability. Further examination of this trend showed that PC1 flammability was negatively correlated with total waxes (i.e., higher flammability; r = −0.51; p = 0.05), particularly with non-alkane compounds (r = −0.55; p = 0.03), and within this group with ketones (r = −0.56; p = 0.03). Among these, 16-hentriacontanone exhibited the strongest relationship (r = −0.61; p = 0.01), although this compound was not present in all species or samples. When PC1 flammability was further examined, these relationships were mainly driven by negative correlations between total waxes—especially non-alkanes and ketones, including 16-hentriacontanone—and ignition time and combustion time (**Supplementary Figure A3**). The same pattern was observed for terpenoids, particularly triterpenes, which were also negatively correlated with ignition time (r = −0.53; p = 0.04) and combustion time (r = −0.59; p = 0.02).

Among alkane compounds, and despite being scarcely registered, tricosane showed a positive correlation with flammability variables. Higher tricosane concentrations were associated with higher PC1 flammability values (i.e., lower flammability; r = 0.88; p < 0.001). This pattern was reflected in lower values of probability of ignition and flame duration, and higher values of ignition time and combustion time. Additional significant relationships included a negative correlation between hexacosane concentration and combustion time (r = −0.57; p = 0.02), and a positive correlation between nonacosane concentration and flame duration (r = 0.554; p = 0.03), suggesting that higher nonacosane concentrations are associated with longer-lasting flames.

## 4 Discussion

Our results are consistent with the hypothesis that specific chemical compounds contained in cuticular waxes are associated with different patterns of leaf flammability. Across the analyzed species of the Chilean sclerophyll forest, chemical characterization revealed diverse compounds, including alkanes, alcohols, aldehydes, esters, ketones, olefins, tocopherols, and triterpenes, highlighting an interspecific variability that can be associated with species chemical fingerprint. As expected, alkanes were detected in all species, confirming their role as fundamental components of cuticular waxes in vascular plants, particularly in evergreen sclerophyllous trees and shrubs, where chain lengths typically range from C21 to C35 (Samuels et al., 2008; Ormeño et al., 2020). However, in contrast to previous work that primarily focused on alkanes, specifically on litter flammability (Ormeño et al., 2020), our results suggest that flammability patterns of live fuels were not associated exclusively with alkanes. Instead, non-alkane compounds showed strong associations with flammability metrics, particularly ignition time and combustion duration. Notably, non-alkane compounds represented a substantial proportion of total waxes extracted in four of the five chemically analyzed species (72% on average). Nonetheless, because our methodology considered partial pooling of individual hexane leaf extracts in order to obtain representative species-level chemical profiles while minimizing the influence of individual variation, correlations should be interpreted as relationships between species-level chemical profiles and corresponding patterns in flammability with limited ability to resolve intraspecific chemical variation.

A substantial body of literature has traditionally emphasized the influence of terpenes on plant flammability, generally associating higher terpene concentrations with increased flammability due to their low flash points and high volatility (Alessio et al., 2008; Ormeño et al., 2009; Pausas et al., 2016). However, studies increasingly acknowledge that terpenes are not the sole chemical drivers of flammability, as multiple interacting chemical and physicochemical traits can modulate plant responses to fire. From a chemical ecology perspective, this has motivated a broader exploration of foliar chemistry beyond terpenes, with recent studies aiming to identify additional compound classes that may help explain the complex chemical underpinnings of plant flammability traits (Guerrero et al., 2024). Within this framework, the present study places particular emphasis on cuticular waxes, as they constitute the outermost chemical interface between plant tissues and an ignition source. By focusing on this often-overlooked chemical barrier from a fire ecology perspective, our results contribute to a more integrative understanding of how leaf surface chemistry can influence flammability. Due to their particular characteristics and beyond their protective functions, leaf waxes have been used as ecological indicators, including, for example, to trace different vegetation sources in soils, reconstruct paleobotanical records, and assess anthropogenic disturbances or organic matter inputs to aquatic systems (Jansen et al., 2006; Lu et al., 2014; Cerda-Peña and Contreras, 2022). Our results contribute to extending this framework by demonstrating that cuticular wax composition may provide useful information for understanding interspecific variation in flammability.

Based on the combined analysis of flammability parameters, the six studied species were consistently classified into high, intermediate, and low flammability categories. Integrating chemical composition with flammability metrics revealed that species characterized by higher concentrations of non-alkane compounds in their cuticular waxes were associated with higher flammability, suggesting that these compounds may contribute to flammability patterns beyond alkanes alone. This pattern may be related to the particularly strong contribution of ketones and triterpenes, which showed negative associations with ignition time in our study. In contrast to the more thermally stable alkane fraction, these compounds may facilitate the formation of volatile combustible products during heating, potentially accelerating ignition. Among the species classified as highly flammable, *P. lingue* and *C. alba* were characterized by shorter ignition times, shorter combustion durations, and ignition frequencies reaching 100%. Both species exhibited the highest total wax loads and similar proportions of multiple compound classes (alkanes, aldehydes, alcohols, esters, ketones, and terpenoids), probably reflecting a shared phylogenetic signal within the Lauraceae family. Notably, both species displayed the highest concentrations of ketones among the analyzed species, with 16-hentriacontanone being especially abundant in *P. lingue*, the species showing the shortest ignition time between the two species. Previous studies (Guerrero et al., 2021, 2024) also identified this compound as characteristic of highly flammable species (including *C. alba*), suggesting that it may be associated with increased flammability in these species. The low flash point of 16-hentriacontanone (35.6 °C) (Rumble, 2025), together with potential synergistic interactions with other cuticular compounds, may be associated with enhanced ignition, especially considering that cuticular waxes constitute the first interface exposed to flames or a heated atmosphere.

*Escallonia pulverulenta* was also classified as having a high to intermediate flammability. Although its chemical profile could not be determined, this study provides the first flammability assessment for this species, representing valuable information for fire management. *Lithraea caustica*, classified as intermediate flammable, was characterized by a high diversity of chemical compounds in its cuticular waxes, with a predominance of alkanes (mainly hentriacontane and nonacosane) along with elevated concentrations of esters and aldehydes. Although previously reported as weakly flammable (Guerrero et al., 2024), its intermediate position here, together with the detection of 16-hentriacontanone (although at lower concentrations than in *P. lingue* and *C. alba*), supports a context-dependent role of wax composition. This also highlights that flammability rankings are relative to the species set analyzed. *Quillaja saponaria* also showed intermediate flammability and was characterized by a low total wax content and a predominance of alkanes with low concentrations of non-alkane compounds. Although its chemical profile differed from that of *L. caustica*, both species showed the highest concentrations of nonacosane, with levels two to four times higher than those observed in the other species. *Cryptocarya odorifera* clustered as the least flammable species, principally exhibiting the longest ignition times. Ignition frequency of *C. odorifera* was remarkably low, with only 30% of the samples igniting. This species showed low total wax content and reduced chemical diversity, similar to that observed in *Q. saponaria*. However, while *Q. saponaria* was dominated by alkanes (hexacosane, octacosane, and nonacosane), *C. odorifera* exhibited a relatively higher proportion of non-alkane compounds, particularly aldehydes and ketones. Despite this, *C. odorifera* had more than 2.5-fold lower total wax content and over threefold lower non-alkane concentrations compared to *P. lingue* and *C. alba*. Hence, the low ignition probability observed in *C. odorifera* may be associated with both its relatively low total wax content and its specific chemical composition, suggesting that overall wax load and the relative abundance of different compound classes may jointly contribute to the observed flammability patterns. Among alkanes, heptacosane, octacosane, and nonacosane were consistently identified in the cuticular waxes of all species analyzed, with the highest concentrations observed for nonacosane. These compounds have been reported at high concentrations in Mediterranean herbs, shrubs, and tree species (Martins et al., 1999; Norström et al., 2017; Ormeño et al., 2020), as well as in dominant species of the Chilean Mediterranean ecosystem previously evaluated (Guerrero et al., 2021, 2024). Because quantification was based on 1-octadecanol as an external standard, the concentrations reported here should be considered semi-quantitative estimates, although the predominance of the long hydrocarbon chain in the compounds analyzed is expected to result in a small error only, due to the relatively similar GC-MS response factors. In the present study, nonacosane was the dominant alkane in *Q. saponaria*, exhibiting a strong predominance similar to that reported for evergreen Mediterranean oaks such as *Quercus* suber or *Q. ilex* (Martins et al., 1999), and other species from the Andean temperate forest, including, for example, *Araucaria araucana* or *Nothofagus dombeyi* (Cerda-Peña and Contreras, 2022). From an ecological perspective, the predominance of long-chain n-alkanes such as nonacosane may be related to their role in limiting cuticular water loss, a particularly relevant function under the seasonally dry conditions of Mediterranean-type ecosystems (Bourdenx et al., 2011; Ormeño et al., 2020). Thus, its high relative abundance in *Q. saponaria* may primarily reflect an ecophysiological role in water conservation rather than a specific adaptation to fire. The ubiquitous presence of these compounds across all species examined reflects their fundamental role as long-chain alkanes in the structural organization of cuticular waxes. While these compounds are typically associated with cuticular functions such as water regulation, our results indicate that nonacosane is positively related to flame duration, suggesting a role in the sustainability component of flammability (Pausas et al., 2017). This finding is particularly relevant because, unlike terpenes whose concentrations are highly variable, hydrocarbons such as nonacosane represent structurally stable and ubiquitous components of leaf surfaces across most species in the region. Nevertheless, our results indicate that, considering cuticular waxes, it is the combination of these structurally stable compounds with other more chemically reactive constituents (such as ketones and aldehydes) that ultimately may modulate fire risk.

In line with our initial hypothesis exploring the possible associations between cuticular waxes and flammability, our results indicate that the role of alkanes in flammability appears complex and strongly context-dependent. While alkanes appear to contribute positively to certain flammability components, their influence on tissue ignitability seems to depend on their interaction with other previously hidden chemical groups, such as ketones or terpenoids. Alkanes are known to be highly resistant to degradation, maintaining their chemical structure over time. It is therefore plausible that discrepancies with previous studies (e.g.; Ormeño et al., 2020), focused on litter flammability, arise because live foliage contains higher concentrations and greater variability of reactive compounds in contrast to senescent litter material. Future studies comparing fresh leaves and litter flammability together with chemical profiles would allow further testing of this possible explanation.

Overall, our findings suggest that variation in cuticular wax composition is associated with differences in leaf flammability among species. In particular, non-alkane compounds, especially ketones and terpenoids, showed consistent associations with several flammability metrics. Although the limited number of species evaluated prevents establishing causal relationships, these results highlight cuticular wax chemistry as a promising and previously underexplored component of plant flammability, particularly considering live foliage. Future studies including a broader range of species and experimental approaches will be necessary to clarify the mechanisms underlying these associations and to evaluate their generality across Mediterranean-type ecosystems. Furthermore, other leaf traits not measured in this study, such as leaf thickness, specific leaf area, and surface area-to-volume ratio, may covary with both cuticular wax deposition and flammability, potentially contributing to the observed associations. Evidence from previous research, including leaf flammability studies, suggests that certain plant water-use strategies aimed to reduce water loss and prevent desiccation, may also contribute to lower flammability (Favero et al., 2026). While some cuticular wax compounds have low flash points and may promote ignition and increase combustibility, these same waxes can influence desiccation rates by reducing moisture loss, thereby decreasing ignitability. This potential dual role opens new avenues for research, providing a foundation for future studies aimed at disentangling these opposing mechanisms and contrasting effects. This is particularly relevant in the context of ongoing climate change, where increased investment in traits that reduce water loss may simultaneously influence plant vulnerability to fire.

Although cuticular waxes represent a relatively small fraction of total leaf mass, their location at the outermost leaf surface places them at the first chemical interface exposed to thermal radiation. Their potential contribution to ignition should therefore be considered together with, rather than as overriding, bulk leaf properties such as fuel moisture content. In our experiment, fuel moisture content was relatively high (~133%) but did not differ significantly among species. However, because FMC was determined from a separate subsample rather than from the same leaf material used in the flammability assays, its relationship with flammability should be interpreted cautiously, and these results do not imply that moisture was unimportant in determining live-leaf flammability. Importantly, these relationships were evaluated at the leaf scale under controlled experimental conditions. The epiradiator assay provides standardized thermal exposure and is therefore useful for comparing intrinsic leaf-level flammability among species; however, it does not reproduce the complexity of field-scale wildfire behavior, where heat flux, flame contact, wind, fuel arrangement and continuity, canopy structure, and interactions among fuel strata can strongly influence combustion. Therefore, the associations identified here should not be directly extrapolated to whole-plant, stand, or landscape-scale fire behavior. Future studies integrating leaf-level chemical traits with larger-scale experimental burning will be necessary to determine whether these relationships persist across organizational scales.

## 5 Conclusions

Our study provides evidence that non-alkane compounds in cuticular waxes are associated with variation in foliar flammability among species. While alkanes are ubiquitous components of plant cuticles and can contribute to flammability, our results indicate that non-alkane compounds were strongly associated with some leaf flammability metrics. Cuticular waxes emerged as a potentially important component associated with foliar flammability in native species of the Chilean sclerophyllous forest. Flammability was associated with both the concentration and chemical composition of cuticular waxes, with each species exhibiting a distinct chemical profile composed of a diverse array of compounds, including alkanes, alcohols, aldehydes, esters, ketones, and terpenoids such as tocopherols and triterpenes. Higher concentrations of cuticular waxes were positively associated with increased flammability, particularly through shorter ignition times and longer combustion, with some non-alkane compounds (i.e., ketones and triterpenes) showing stronger associations with flammability parameters. Hence, beyond total wax concentration, the qualitative composition and specific combination of chemical compounds may contribute to differences in flammability among species.

The Chilean Mediterranean ecosystem, recognized as a global biodiversity hotspot, is currently under severe pressure, mainly from land-use change and prolonged drought periods, conditions that have intensified wildfire frequency and severity over recent decades. Under this scenario, advancing our understanding of chemically driven flammability traits in native species is increasingly urgent. Given the tight link between cuticular waxes and essential physiological functions such as water-loss regulation, functions that are likely to become even more critical under projected climate-driven increases in drought, investigating their chemical composition provides a fundamental scientific basis for fire ecology. Future research should investigate the potential synergistic interactions between cuticular wax chemistry and flammability, as well as their implications for fire-risk forecasting and management. Integrating chemical traits into fire-risk assessment models represents a promising avenue for building more fire-resilient landscapes in Mediterranean-type ecosystems.

## Data Availability

The original contributions presented in the study are included in the article/**Supplementary Material**. Further inquiries can be directed to the corresponding author.
