# Supplementary material for: Hidden fuels: exploring the association between cuticular waxes and leaf flammability in a mediterranean-type forest
Source: Front Plant Sci. 2026 Sep 9;17:1942980. doi: 10.3389/fpls.2026.1942980 (PMC13597931; doi:10.3389/fpls.2026.1942980)
Supplement: Supplementary file 1 [file SupplementaryFile1.docx]

Supplementary Material

# 1 Appendix A. Supplementary data

# 2

# 3 Fig. A1. Pearson correlation matrix among flammability variables (PI, probability of ignition; IT, ignition time; FD, flame duration; CT, combustion time) and fuel moisture content, considering the six native species studied. Significant correlations (p < 0.05) are boxed.

# 6 Fig. A2. Principal components analysis (PCA) of four measured variables, including: probability of ignition (PI), ignition time (IT), flame duration (FD), combustion time (CT), considering the individuals of species analyzed from the Chilean sclerophyll forest, but excluding E. pulverulenta and averaging individuals according to the three composite samples settled for chemical compounds analysis. Symbols in the graph indicate each individual, while species labels indicate the mean species groups’ position in the spatial ordination, including convex hulls for each species.

# 8 Fig. A3. Correlation matrix showing Pearson’s correlation coefficients among flammability variables (PC1 flammability, PI: probability of ignition, IT: ignition time, FD: flame duration, and CT: combustion time), moisture content, and general chemical compound groups, including PC1 chemistry, for five species from the Chilean sclerophyll forest. Significant correlations (p < 0.05) are boxed.

# Fig. A4. Correlation matrix showing Pearson’s correlation coefficients among flammability variables (PC1 flammability, PI: probability of ignition, IT: ignition time, FD: flame duration, and CT: combustion time), moisture content, and alkane compounds, for five species from the Chilean sclerophyll forest. Significant correlations (p < 0.05) are boxed.
